# Evaluation of Blast Resistance in Zinc-Biofortified Rice

**DOI:** 10.3390/plants14132016

**Published:** 2025-07-01

**Authors:** Anita Nunu, Maina Mwangi, Nchore Bonuke, Wagatua Njoroge, Mwongera Thuranira, Emily Gichuhi, Ruth Musila, Rosemary Murori, Samuel K. Mutiga

**Affiliations:** 1 Kenya Agricultural and Livestock Research Organization—Industrial Crops Research Institute, Mwea, Kerugoya P.O. Box 298-10300, Kenya; gichuhiemily@gmail.com (E.G.); ruthmusila@gmail.com (R.M.); 2 Department of Agricultural Sciences and Technology, Kenyatta University, Nairobi P.O Box 43844-00100, Kenya; maina.mwangi@ku.ac.ke; 3 Department of Plant Sciences, Kenyatta University, Nairobi P.O. Box 43844-00100, Kenya; bonuke.shem@ku.ac.ke; 4 West Africa Centre for Crop Improvement (WACCI), College of Basic and Applied Sciences, University of Ghana, Legon, Accra P.O. Box LG 25, Ghana; nwagatua@wacci.ug.edu.gh; 5 Kenya Agricultural and Livestock Research Organization—Horticulture Research Institute (HRI), Thika P.O. Box 220-01000, Kenya; dmwongerathuranira@gmail.com; 6 International Rice Research Institute (IRRI), IRRI Africa Regional Office, Nairobi P.O. Box 30709, Kenya; r.murori@irri.org; 7 Biosciences Eastern and Central Africa-International Livestock Research Institute (BecA-ILRI), Nairobi P.O. Box 30709, Kenya; 8 Department of Botany, School of Physical and Plant Sciences, Maseno University, Maseno P.O. Box Private Bag, Kenya

**Keywords:** rice breeding, zinc biofortification, blast disease, host resistance, zinc foliar application, anthocyanin

## Abstract

Rice is a staple food for over half of the world’s population, and it is grown in over 100 countries. Rice blast disease can cause 10% to 30% crop loss, enough to feed 60 million people. Breeding for resistance can help farmers avoid costly fungicides. This study assessed the relationship between rice blast disease and zinc or anthocyanin content in biofortified rice. Susceptibility to foliar and panicle blast was assessed in a rice panel which differed on grain zinc content and pigmentation. A rice panel (n = 23) was challenged with inoculum of two isolates of *Magnaporthe oryzae* in a screenhouse-based assay. The zinc content with foliar blast severity was analyzed in the leaves and grain of a subset of non-inoculated rice plants. The effect of foliar zinc supplementation on seedlings was assessed by varying levels of zinc fertilizer solution on four blast susceptible cultivars at 14 days after planting (DAP), followed by inoculation with the blast pathogen at 21 DAP. Foliar blast severity was scored on a 0–9 scale at 7 days after inoculation. The rice panel was scored for anthocyanin content, and the data were correlated with foliar blast severity. The panel was grown in the field, and panicle blast, grain yield and yield-related agronomic traits were measured. Significant differences were observed in foliar blast severity among the rice genotypes, with IRBLK-KA and IR96248-16-2-3-3-B having mean scores greater than 4, as well as BASMATI 370 (a popular aromatic variety), while the rest of the genotypes were resistant. Supplementation with foliar zinc led to a significant decrease in susceptibility. A positive correlation was observed between foliar and panicle blast. The Zn in the leaves was negatively correlated with foliar blast severity, and had a marginally positive correlation with panicle blast. There was no relationship between foliar blast severity and anthocyanin content. Grain yield had a negative correlation with panicle blast, but no correlation was observed between Zn in the grain and grain yield. This study shows that Zn biofortification in the grain may not enhance resistance to foliar and panicle blast. Furthermore, the zinc-biofortified genotypes were not agronomically superior to the contemporary rice varieties. There is a need to apply genomic selection to combine promising alleles into adapted rice genetic backgrounds.

## 1. Introduction

Rice (*Oryza sativa* L.) is a staple food crop for more than half of the world’s population [1]. It plays a key role in providing food security, income and employment opportunities for many people. In Kenya, the demand of rice is higher (approximately 1,200,000 tons) than its production (230,000 tons) and the country relies on imports to bridge the gap [2]. This demand is constantly increasing due to the urban lifestyle and changing food preferences, which leads to the adoption of easy and quick to cook foods that require less energy [3]. To achieve rice sufficiency, and food security in Kenya, there is a need to adopt strategies which will overcome the many constraints to production, including those that are related to climate change.

Rice production in Kenya is affected by multiple constraints such as diseases, pests (insects, rats, birds and molluscs), saline soils, and soil nutrient deficiency [2]. Rice blast disease, caused by a filamentous hemiobiotophic fungal pathogen called *Magnaporthe oryzae B.C. Couch*, is one of the major biotic constraints in Kenya [4]. The symptoms exhibited and severity of the disease depend on the strain and the environmental conditions in which the host crop grows [5]. This devastating disease attacks multiple plant parts, causing symptoms on the leaves, the neck and the panicle [6]. The typical blast disease symptoms include diamond-shaped lesions with greyish or whitish centers and dark-brown borders on the leaves, necrosis on the neck tissue, and whitish or greyish panicles with empty spikelets [7]. The occurrence of the disease is favored by hot humid weather, and it is spread by wind, insects or flooding [7]. Agronomic practices such as high application of mineral nitrogenous fertilizer have been reported to favor blast disease [8]. Although the disease was first reported in the 18th century in Asia, rice blast was first reported in Kenya in 2008 [4]. The rice-producing regions of Kenya experienced about 80% grain yield loss due to a blast disease outbreak in 2009 [4]. Although the disease can be controlled by fungicide sprays, the chemical control method is unaffordable to most of the small-scale growers, and it is harmful to the environment. Breeding for blast-resistant rice varieties can be a more sustainable approach to reducing yield loss due to the damage caused by *M. oryzae*.

The plant disease resistance arsenal is modulated through biochemical pathways, which utilize mineral elements to produce compounds which enhance pathogen effector recognition or the formation of protective layers by the host plant [9]. Certain mineral elements directly influence plant health by modulating the formation of defense compounds such as ethylene, jasmonic acid, salicylic acid, and phytoalexins, or physical barriers such as callus and/or lignin, or through alterations in the plant microflora, the rhizosphere nutrient content, and the pH [9,10,11]. Some mineral elements are useful in enhancing plant host resistance and are also useful for human health [12]. Reports indicate that there are optimal rice grain zinc concentrations which could have health benefits for consumers, particularly in enhancing immunity and hence preventing diseases such as cancer [13], type I and type II diabetes [14], hair and memory loss, skin problems, weakening of body muscles, infertility in men, and pneumonia in children [15]. Rice grain contains energy and many other essential nutrients for human intake, but it lacks zinc at levels high enough for optimal health [16]. Besides its importance in human health, the mineral zinc is known to reduce the sterility of rice spikelets, and its application has also been associated with reduced damage by rice blast and sheath blight diseases [17,18]. Studies have shown that zinc influences fungal disease severity by enhancing the release of antioxidant enzymes such as superoxide dismutase, activation of pathogenesis related (PR) genes and intensification of the lignin cell walls that act as physical barriers against pathogen entry and colonization [17,18]. On the other hand, antioxidants are also known to protect humans against diseases. Anthocyanin is an antioxidant whose content in rice is reported to possess many functional properties relevant to human health, including protection of endothelial cells, prevention of heart and cardiovascular disease, anti-cancer activity, control of diabetes, and amelioration of eyesight [19,20].

Improving grain Zn and anthocyanin contents in rice crops could, therefore, be a promising avenue for producing more nutritious food, which can reduce malnutrition among rice consumers [16,20]. The Kenya Agricultural and Livestock Research Organization (KALRO) has collaborated with the International Rice Research Institute (IRRI) in breeding for Zn-biofortified rice. The current study was conducted to complement the ongoing regional breeding efforts for blast resistance and zinc biofortification in rice. The objectives of this study were to investigate the following aspects of the rice blast pathosystem: (1) whether zinc biofortification affected susceptibility to rice blast, (2) whether foliar supplementation of zinc influenced blast severity, (3) whether anthocyanin content influenced blast severity, and (4) whether the amount of zinc in the leaves or the grain was correlated with blast severity, key agronomic traits, and rice grain yield.

## 2. Results

### 2.1. Response of the Rice Genotypes to Inoculation with Two Isolates of Magnaporthe oryzae

**Figure 1:**
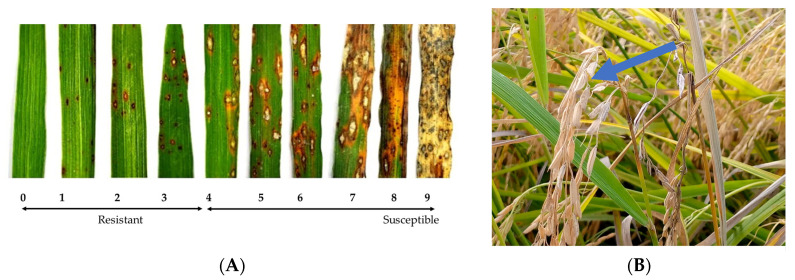
Rice blast disease symptoms. (**A**) Foliar blast severity score scale based on percentage of damaged leaf area, and (**B**) typical panicle blast shown by a blue arrow. The blast severities for the foliar and panicle blasts were scored on a 0–9 scale following the standard evaluated system established by IRRI [21]. The panicle blast severity score scale was as follows: 0 = no visible or observed lesions on only a few pedicels; 1 = lesions on several pedicels or secondary branches; 3 = lesions on a few primary branches or the middle part of panicle axis; 5 = lesions partially around the base (node) or the uppermost internode or the lower part of panicle axis near the base; 7 = lesions completely around the panicle base or uppermost internode or panicle axis near base with more than 30% of field grains; 9 = lesions completely around the panicle base or uppermost internode or the panicle axis near the base with less than 30% of filed grains.

**Figure 2:**
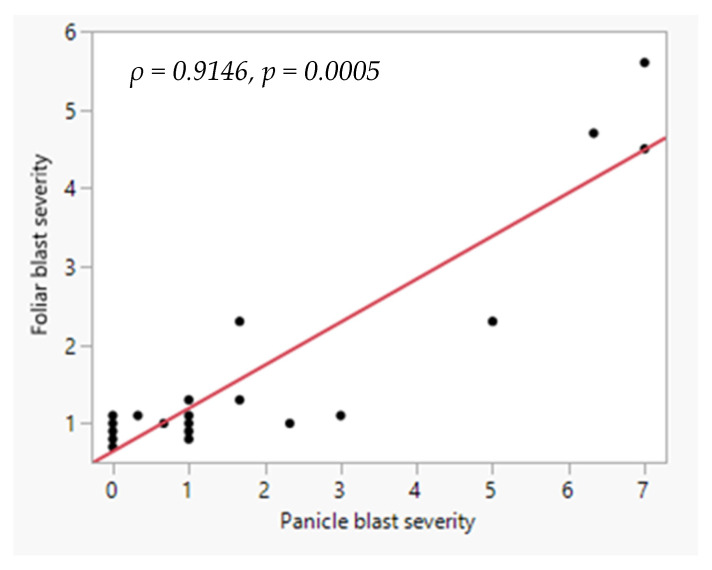
Relationship between foliar blast and panicle blast severity in twenty-three rice genotypes which were evaluated at Mwea Research Station, Kenya. The foliar blast was scored on 28-day-old seedlings which had been inoculated with two blast pathogen isolates from the rice-growing parts of Kenya. Panicle blast severity was scored on plants which had been grown and naturally infected by the resident field blast pathogen at Mwea. Blast severity was scored on a scale of 0–9, where 0 = no visible damage on the leaves or panicles; 9 = visible damage on more than 75% of the leaf or panicle tissue.

The response of the genotypes was assessed based on foliar blast scores obtained from inoculations under a controlled environment and the panicle blast scores obtained from rice, which was naturally infected with the resident inoculation. The analysis showed a strong positive correlation between the foliar and panicle blast data (*ρ* = 0.91, *p* = 0.0005; Figure 1 and Figure 2). Inoculation of the rice seedlings with the two isolates, without foliar zinc application, did not cause differing disease reactions on the rice genotypes (*χ*^2^ = 0.99960, *df* = 1, *p* < 0.0001). The isolates did not cause disease symptoms on at least 87% of the tested rice genotypes (Table 1).

**Figure 3:**
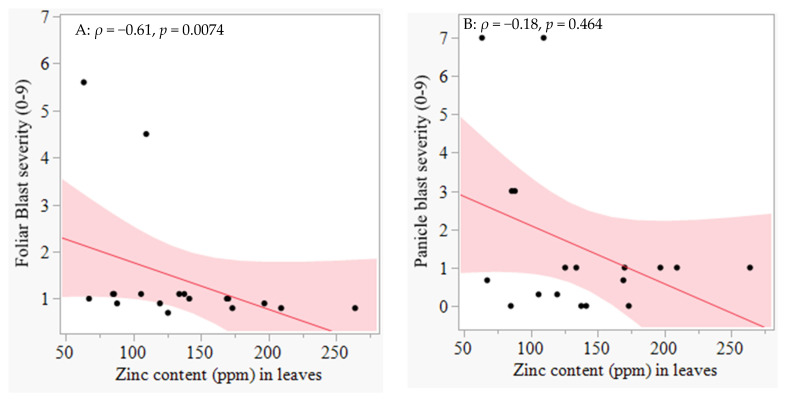
Relationship between zinc in the leaves and blast severity in twenty-three rice genotypes which were evaluated at Mwea Research Station, Kenya. (**A**) Foliar blast severity, (**B**) panicle blast severity. Foliar blast was scored on 28-day-old seedlings which had been inoculated with two blast isolates from the rice-growing parts in Kenya. Panicle blast severity was scored on plants which had been grown and naturally infected by the resident field blast pathogen at Mwea. Blast severity was scored on a scale of 0–9, where 0 = no visible damage on the leaves or panicles; 9 = visible damage on more than 75% of the leaf or panicle tissue. Zinc content in the leaves was analyzed on a sub-set of the twenty-three rice genotypes of 21-day-old seedlings which had not been inoculated with blast isolates.

The isolate KE0002 was found to attack BASMATI 370 and IR 96248-16-2-3-3-B, and not the other twenty-one rice genotypes. Similarly, KE0215 successfully caused disease only on five (BASMATI 370, IRBLK-KA, IR 96248-16-2-3-3-B, IR 99636-93-3-3, and IR10M210) out of the twenty-three rice genotypes tested (Table 1). Although IRBLK-KA did not develop disease symptoms when inoculated with KE0002, it was successfully attacked by KE0215 (Table 1). An analysis of the zinc concentration in the leaves (PPM) showed a negative correlation with foliar blast severity, but no signification association was observed between the panicle blast severity and the foliar zinc content (Figure 3).

However, the amount of zinc in the grain did not correlate with either foliar blast or panicle blast (*ρ* = 0.3734, *p* = 0.1269 and *ρ* = 0.4663, *p* = 0.0511), respectively. A test of the ranking of the rice genotypes based on the zinc concentration of BLUPs showed that the amount of zinc in grains was not correlated with the Zn level in the leaves (*ρ* = −0.15, *p* = 0.5412; Table 2). When the graduated scale for pigmentation was used in an analysis for the effect of anthocyanin, no significant effects were observed in genotypes of varying anthocyanin levels. However, grouping of the rice genotypes into those with or without visible pigmentation showed that purple-pigmented rice was three times more likely to be attacked by blast than white rice (OR = 2.98, *χ*^2^ = 21.26, *df* = 1, *p* < 0.0001).

### 2.2. Blast Disease Response of Rice Genotypes with Foliar Zinc Fertilizer Application

Differences in blast severity were observed among the four rice genotypes which had received varying levels of zinc fertilizer (*p* < 0.0001). Although the severity differed, all the rice genotypes remained susceptible to blast as the severity scores were greater than 5 (Table 3). Furthermore, blast severity differed among the zinc levels that were applied on the rice genotypes (*p* = 0.041). Interestingly, increasing the concentration of the foliar zinc fertilizer was associated with a decrease in foliar blast severity but all the severity scores were above 5 (on a scale of 0–9; Table 4). No significant interaction was observed between the zinc level and the rice genotype (*p* > 0.05). A binomial logistic regression showed that neither the rice genotypes (χ^2^ = 9.9036, *df* = 3, *p* > 0.0194) nor the zinc application rates (*χ*^2^ = 8.7318, *df* = 5, *p* > 0.1203) differed in the presence or absence of blast disease.

### 2.3. Agronomic Traits and Their Correlation with the Severity of Panicle Blast Under Field Conditions

Differences in agronomic traits were observed among the twenty-three genotypes grown during the main season at Mwea (Table 5 and Table 6). The flowering time ranged from 75 days in IRBLTA 2-RE to 97 days in BW196 (Table 5). The majority (73.9%) of the rice genotypes flowered later than the check variety, BASMATI 370. All the genotypes had varied plant heights. The shortest variety was IR 99637-123-1-3, at a height of 93 cm, while the tallest variety was IR 96248-16-2-3-3-B, with a height of 122 cm at maturity. A majority (95.7%) of the rice genotypes were shorter than the check variety (Table 5). The highest number of tillers per plant were observed in BW196, with a majority of the genotypes exhibiting more tillers than the popular commercial variety, BASMATI 370. Similarly, the panicle number per plant was highest in BW196, suggesting a positive correlation between tiller number and panicle number per plant. The 1000-grain weight ranged from 19.2 g in IR 96248-16-2-3-3-B to 28 g in BW196 (Table 5). Consequently, the highest number of panicles/plant and heaviest grains observed in BW196 resulted in the high grain yield (5.95 t ha^−1^) observed in BW196. On the other hand, BASMATI 370 exhibited the lowest grain yield of 1.02 t ha^−1^. The panicle length did not differ significantly (*p* > 0.05) among the 23 genotypes (Table 5).

Based on the field evaluation of the rice panel, the typical panicle blast symptoms were observed in IRBL3-CP4, IRBLK-KA, IR 96248-16-2-3-3-B and BASMATI 370, while the rest showed hypersensitive reactions. Panicle blast was negatively correlated with grain yield (*r =* −0.4922, *p =* 0.0076), flowering time (*r =* −0.4774, *p =* 0.0015) and thousand-grain weight (*r =* −0.4986, *p =* 0.0078). However, panicle blast was positively correlated with plant height (*r =* 0.5433, *p =* 0.0750). Panicle blast was not correlated with panicle length or the number of tillers per plant (Table 6). Interestingly, grain yield was positively correlated with the amount of zinc in the leaves (*ρ* = 0.3231, *p* = 0.1908) but did not correlate with the amount of zinc in the grain ((*ρ* = 0.0361, *p* = 0.8869).

### 2.4. Agronomic Traits and Their Correlation with Foliar Blast Severity

**Figure 4:**
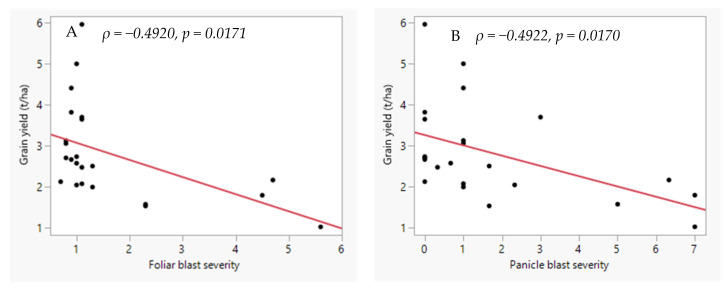
The relationship between blast severity and grain yield (t ha^−1^) of the twenty-three rice genotypes which were evaluated at Mwea Research Station. (**A**) Foliar blast, (**B**) panicle blast. Foliar blast severity was scored on 28-day-old seedlings which had been grown in a screenhouse. Panicle blast severity was scored on plants which had been grown and naturally infected by the resident field blast pathogen at Mwea. Blast severity was scored on a scale of 0–9, where 0 = no visible damage on the leaves or panicles; 9 = visible damage on more than 75% of the leaf or panicle tissue.

Foliar blast severity was negatively correlated with grain yield (*ρ* = −0.49, *p* = 0.00598) and flowering time (*ρ* = −0.4608, *p* = 0.0269; Figure 4). On the other hand, foliar blast had a significant positive correlation with plant height (*ρ* = 0.5402, *p =* 0.0361; Table 6). Foliar blast did not have a significant correlation with either panicle length, thousand-grain weight, number of tillers, or panicles (Table 6).

## 3. Discussion

Breeding for blast resistance in zinc-biofortified rice variety can be a huge success in enhancing food nutrition and security African communities. In this study, a panel of rice from a zinc biofortification breeding project was tested for response to blast pathogen attack under controlled and field conditions in Mwea, Kenya. Although the major goal of this study was to investigate whether foliar or grain zinc content can influence the susceptibility of rice to blast disease, the complementary role of anthocyanin was also evaluated. Additionally, the relationships between blast disease severity and rice agronomic traits were assessed. To the best of our knowledge, this is the first study which assesses the combined effects of zinc and anthocyanin content on rice blast disease. The findings provide insights into the role of these nutrients and the potential blast management strategies for Africa and beyond.

Based on the screenhouse inoculations, the two isolates of *M. oryzae* did not differ significantly in disease reactions. The lack of differences could imply that the isolates had a similar virulence spectrum, with similar effectors being used to attack the genetically distinct rice genotypes. Each of the isolates originated from a different rice-growing zone in Kenya. Although these isolates have been characterized through inoculations on varying rice genotypes, revealing that KE0215 is more virulent than KE0002, a detailed effector profiling has not been conducted using a shared platform for both isolates [22]. There is ongoing research to identify effectors based on whole-genome sequencing of KE0002. Thus, there is a need to genotype the pathogen collections using a similar platform and to utilize more than one isolate from each of the target rice-growing regions for blast resistance screening.

The current study showed significant genotypic differences in foliar and panicle blast severity. For foliar blast, most of the zinc-biofortified genotypes did not show a susceptible reaction against the two isolates. However, IR 96248-16-2-3-3-B had a susceptible reaction against the two isolates, while IR 10M210 and IR 99636-93-3-3 had typical blast symptoms when inoculated with KE0215. There was no information about whether these genotypes harbored any of the known blast resistance genes. Inoculation of IRBLTA 2-RE (a monogenic line with *Pita-2* R- gene) did not show typical blast symptoms against any of the two isolates of *M. oryzae*. Inoculation of IRBLK-KA (*Pik*), IRBL11-Zh (*Pi-11*) and IRBL3-CP4 (*Pi3*) showed a susceptible reaction against at least one of the isolates. A previous study reported that at least 80% of the tested isolates from East Africa could not cause disease symptoms on IRBLTA 2-RE and IRBL11-Zh [23]. On the other hand, a high susceptibility rate (≥95%) had been reported for IRBLK-KA, IRBL3-CP4 and IRBTP16211 [23]. The IRBLs were bred into IRBTP16211 (LTH), which was presumed to be universally susceptible but has since been shown to harbor the *Pik-1 R* gene, which could be effective against some blast pathogen collections [23]. The genotypes with a susceptible reaction for panicle blast included IRBL3-CP4, IRBLK-KA, IR 96248-16-2-3-3-B and BASMATI 370. These findings confirm the susceptibility of IR96248-16-2-3-3-B and BASMATI 370 to the two types of blast disease, and that *Pita-2* could be conferring resistance to most *M. oryzae* pathogen strains in Kenya. It should be noted that BASMATI 370 is a popular aromatic commercial cultivar which has been reported to be highly susceptible to blast [22,23]. Testing the zinc-biofortified rice panel with a wider set of isolates which represent the disease virulence strengths based on known virulence genes could give detailed insights into the resistance spectrum of the panel.

Purple-pigmented rice was three times more likely to have a susceptible disease reaction compared to white rice. The observed association between rice pigmentation and blast disease severity was unexpected because anthocyanins have been documented to play a great role in enhancing plant immunity [24]. For example, anthocyanins have been proposed to serve as “sunscreens” for extreme weather and as antioxidants which scavenge reactive oxygen species to guard the plants against abiotic and biotic stresses [25,26]. Indeed, pigmented rice was found to be more resistant to bacterial leaf blight compared to white rice [27]. The purple-pigmented rice genotype was expected to be more tolerant to foliar blast disease severity as compared to the white-pigmented rice. It should be noted that the core focus of this study was not on the role of pigmentation on blast resistance, but this was evaluated as a complementary trait. We acknowledge the lack of a wider range of pigmentation on the rice genotypes which could have given more insights on the relationship between the two important traits. To obtain a more reliable result, further studies should include a rice panel with a broader scale of anthocyanin content.

Foliar blast had a significant negative correlation with the amount of zinc in the rice leaves, but not with the zinc in the grain. This observation means that characterization for the relationship between blast and Zn should be focused on the leaves because they are the primary tissue showing the typical symptoms of the disease. Here, the amount of Zn in the leaves was determined on 21-day-old seedlings, while Zn in grains was quantified on the mature harvested grain after harvesting. The zinc absorbed from the soil by the roots or through stomata on the plant surface is used in the biosynthesis of simple and complex molecules within the leaves [28]. The synthesized compounds are translocated to different sink organs, where they are utilized in growth, metabolic activities, plant defense, etc. The amount of zinc which accumulates in the young leaves is later translocated to the flowers and the grain [17,28]. Recent studies show that 43% of the zinc applied as a foliar supplement was translocated to the grain [29]. Owing to the nature of allocation of the micronutrients under optimal Zn in the soil, young plant leaves will always have more zinc than mature grain [28]. Upon translocation of zinc from the roots into the leaves, the latter becomes the source of the nutrient for other plant organs, including the leaves and grains [28]. The amount of zinc which is translocated for subsequent use or storage within the seed is not involved in resistance to blast. The mechanisms through which zinc enhances disease resistance in plants have been reviewed [10,17,30]. Zinc is a cofactor for antioxidant enzymes such as superoxide dismutase, which has been shown to enhance plant disease resistance through detoxification of the harmful level of reactive oxygen species upon infection [17]. Zinc is involved in the signaling of defense pathways, such as salicylic and jasmonic acid [17]. Furthermore, Zn is also known to contribute to the synthesis of lignin and callose, compounds which act as barriers to fungal penetration into the host cells, and provides the general vigor of the plant by supporting chlorophyll production, photosynthesis, and regulating the synthesis and activity of proteins [17,30].

Typical blast lesions were formed on susceptible rice cultivars treated with the zinc fertilizer and inoculated with the pathogen, revealing that zinc did not induce resistance. However, increasing zinc may have strengthened the cell wall, delaying the penetration of the fungus. Many studies have reported that supplemental application of zinc reduces plant symptoms [31,32]. It has been proposed that the zinc-mediated decrease in disease symptoms could be due to direct toxicity to inhibit the growth of the pathogen, scavenging of potentially harmful reactive oxygen species (ROS) produced during the attack of the plant by the pathogen, or by enhancing Zn-triggered organic defenses [10,33,34]. In the current study, the plants showed a susceptible reaction, although a reduction in the susceptibility score was observed. It is possible that increasing Zn above the reported concentration would have resulted in inhibition of the pathogen growth and hence induced resistance, but this was not explored.

With rice becoming a staple food for most Kenyans, the goal of the IRRI-KALRO rice biofortification effort is to boost the nutritional value of rice through breeding for substantial zinc into high-yielding rice varieties. Rice grain yield is correlated with multiple complementary agronomic traits and is modulated by abiotic and biotic stresses. The genotypes evaluated in this study differed in agronomic traits, grain yield and response to blast disease. To advance the breeding effort, there is a need to compare the rice genotypes based on their resilience and grain yield. Reaction to foliar and panicle blasts is a measure of resilience of the genotypes against a major biotic constraint in rice production in Kenya. Based on resistance to blast disease and grain yield, the most promising biofortified genotypes were IR 08M118 and IR 99681-38-1-2, but these were not better than three local varieties (i.e., BW196, Komboka and ARICA 17). Future efforts should focus on integrating important traits to enhance crop resilience and nutritional value through robust methods, such as genomic selection, while mining the promising alleles in the adapted rice genotypes.

## 4. Materials and Methods

### 4.1. Site of This Study

This study was carried out at the Kenya Agricultural and Livestock Research Organization (KALRO), Mwea Research Station, in Kirinyaga County. Mwea Research Station is located about 21 km southwest of Embu town and 112 km northeast of Nairobi City. The altitude of the station is 1159 m above sea level. Mwea experiences a daily average temperature of 22 °C, with a range of 15.6 °C to 28.6 °C. The region has bimodal rain with the long rains of 850 mm falling between March and June, and short rains of 350 mm between October to December.

### 4.2. Rice Germplasm

The panel of twenty-three rice genotypes consisted of four monogenic lines carrying individual blast resistance genes (also known as the international rice blast differential lines (IRBLs)), a universal susceptible japonica cultivar (Lijiangxintuanheigu, LTH—a recurrent parent of the IRBLs), both of which were kindly provided by the Durable Rice Blast Resistance for sub-Saharan Africa project [35], thirteen genotypes from the zinc-biofortified lines bio-fortification project of IRRI, and five local cultivars obtained from Kenya Agricultural and Livestock Research Organization (KALRO) (Table 7).

### 4.3. Fungal Isolates and Preparation of the Inoculum

Two isolates of *Magnaporthe oryzae*, which had been characterized in earlier studies, were selected to represent the spectrum of virulence for the two main rice-growing regions of Kenya [23]. The isolates were stored at −20 °C in dry filter paper at Kandara Research Station of KALRO prior to use in the current studies. The isolates were grown in oatmeal agar for 72 h with 12 h light at 25 °C. Pure mycelial cultures were transferred into rice bran agar for sporulation. The plates with confirmed sporulation were washed with 10 mL of (0.2%) Tween 20 solution under a biosafety cabinet using a soft brush. The mixture was filtered using a cheese cloth into a sterile beaker. The inoculum concentration was standardized to 2 × 10^5^ spores/mL using a Hemocytometer.

### 4.4. Sowing and Inoculation of the Rice Seedlings in a Growth Chamber

The rice seeds were planted on plastic trays (Legacy Lab Africa LTD., Nairobi, Kenya) whose holes had been filled with a sterilized soil from the Tebere rice-growing scheme. The soil was sterilized by autoclaving at 121 °C under 16 psi for 2 h using metal containers. For each rice genotype, ten seeds were randomly sown in each of the three replicates of the trays. The seedlings underwent optimal agronomic management until the 14th day after planting (DAP), when they were applied with varying levels of foliar zinc fertilizer, and/or until 21 DAP, when they were inoculated with KE0215 and KE0002 virulent blast isolates at 21 DAP. The prepared fungal inoculum was sprayed directly on the seedlings using a handheld sprayer. The inoculated seedlings were maintained for 48 h in an incubation polythene chamber whose relative humidity ranged between 95% to 100%. Thereafter, the plants were kept under ambient conditions. Data on foliar blast severity was collected on the 7th day after inoculation following an established score scale of 0–9 (0: no observable symptoms; 1–3: varying degrees of hypersensitive reactions; 4–9: varying intensities of blast severity) [21] from an average of six plants of each genotype (Figure 1).

### 4.5. Preparation and Application of Foliar Zinc Fertilizer

A commercially available foliar fertilizer, King Fol Zinc 70 (70% Zinc; Murphy Chemicals, Nairobi, Kenya), was used as a source of zinc. King Fol Zinc 70 is marketed in Kenya for supplementation of zinc in seedlings of cereals. The recommended application rate is 20 mL to 30 mL in a 20-liter sprayer pump (equivalent to 0.07% and 0.105% zinc, respectively). In this study, foliar fertilizer was diluted for use in treatments consisting of 0% (no zinc control), 0.05%, 0.07%, 0.105%, 0.15%, and 0.20% zinc. The varying Zn fertilizer treatments were applied to 14-day-old seedlings of BASMATI 370, BASMATI217, IRBLK-KA and IR96248-16-2-3-3-B using a handheld sprayer.

### 4.6. Zinc Content Analysis

The amount of zinc was analyzed in the leaves and the grain. Zinc was analyzed in 0.3 g of leaves from 21-day-old seedlings using a previously described Atomic Absorption Spectroscopy protocol, with slight modifications [37]. Briefly, the leaves’ tissue was dried and ground into a fine powder. A digestion mixture (2.5 mL) was added prior to digesting the sample at 110 °C in digester tubes for one 1 h, with addition of 1 mL H_2_O_2_ at three successive cooling steps. The temperature was raised to 330 °C and the heating continued until a colorless solution was obtained. Thereafter, 25 mL of distilled water was added to the solution and stirred to saturation. The mixture was then topped up to 50 mL with distilled water. A sample of the aqueous solution was aspirated in the flame atomizer by the nebulizer and the analyte concentration was measured at a wavelength of 213.9 nm in parts per million (ppm) using model of AAS equipment (model: AA-6200, Shimadzu Kyoto, Japan).

The amount of zinc in rice grain was analyzed in a 5 g sample with a slight modification of a previously reported protocol using an X-ray fluorescence analyzer (model: VMR-CCC-G3-U, Olympus Scientific Solutions America, Waltham, MA, USA) [38]. In this non-destructive method, dehulled grains were added into fluorometric plastic cups and the contents were placed into the spectrophotometer cup holders covered with a transparent plastic film prior to being transferred into the X-ray compartment. Later, the concentrations were read in parts per million (ppm). For a quality check, the equipment was calibrated with the appropriate reference elements for low and high concentrations of zinc prior to a reading of the rice grain samples.

### 4.7. Collection of Agronomic Trait Data

Twenty-three rice genotypes were planted in the field at KALRO Mwea Research Station for evaluation of agronomic traits and panicle blast between the first and the second moths of the main season in 2023. The experiment was laid out in a randomized complete block design, replicated three times, with each plot consisting of 2 rows of 2 m length and a spacing of 20 cm between the rows and 20 cm between the plants. The experiment was established in a lowland-irrigated ecology and the recommended agronomic management practices were applied. Basal NPK fertilizer was applied at a rate of 25 kg ha^−1^ during planting. Two phases of top dressing were thereafter conducted at a rate of 25 kg ha^−1^, with urea in the first phase and sulphate of ammonia in the second phase. Application of insecticides and weeding were carried out at the initial and vegetative stages of the experiment. Data on flowering time were collected based on days to 50% flowering for plants in a plot. At maturity, pre-harvest data were collected on plant height, number of tillers per plant/hill, panicle length and panicle number (number of productive tillers per plant) on a sample of six plants per plot. The entire rice panel was grouped as either pigmented or non-pigmented prior to measuring the anthocyanin content using the photometric method for the pH difference [39]. The post-harvest data were collected on yield-related traits, moisture content, and zinc content accumulated in the grains after harvesting. Data on yield-related traits was used to compute the grain yield.

### 4.8. Scoring of Panicle Blast Severity Data

At the physiological maturity, the entire rice panel was scored for the typical panicle blast symptoms, which are characterized by dark necrotic lesions partially or completely covering the base (node) of the uppermost internode of the lower part of the panicle axis, with partially or unfilled greyish grains (Figure 1). Panicle blast severity was scored following an arbitrary 0–9 score scale established within the standard evaluation system of rice by the International Rice Research Institute (IRRI) [21], where 0 = no visible or observed lesions on only a few pedicels; 1 = lesions on several pedicels or secondary branches; 3 = lesions on a few primary branches or the middle part of panicle axis; 5 = lesions partially around the base (node) or the uppermost internode or the lower part of panicle axis near the base; 7 = lesions completely around the panicle base or uppermost internode or panicle axis near base with more than 30% of field grains; 9 = lesions completely around the panicle base or uppermost internode or the panicle axis near the base with less than 30% of filed grains.

### 4.9. Data Analysis

Data were summarized using Microsoft Excel and analyzed using JMP Pro ver. 18 (SAS Institute Inc., Cary, NC, USA 1989–2023). The effects of rice genotypes, pathogen isolates, anthocyanin, and zinc concentration were tested in a mixed model in which they (the predictor variables) were assumed to have a fixed effect, while replications were random and nested within the pathogen isolates. The experimental means were separated using Tukey’s HSD method at *p* < 0.05. The best linear unbiased predictors (BLUPs) were determined by assigning a random effect on the rice genotypes. Similarly, the predictor variables were included in a binomial logistic regression model for which the significant effects for presence or absence of blast were determined with the binary disease reactions (where disease severity was coded as 0 for scores ranging from 0 to 3, and 1 for scores ranging from 4 to 9) being used as responses. Likelihood ratio tests were used to compare the probable causes for presence or absence of the disease.

## 5. Conclusions

The outputs presented in this study provide insights on the role of foliar and grain zinc and anthocyanin in influencing rice genotypes’ susceptibility to blast disease. The rice genotypes (n = 23) with varying levels of zinc and anthocyanin aided in realizing that the zinc content in the leaves was negatively correlated with foliar blast, and there was no correlation between the anthocyanin pigmentation and blast; this is due to the lack of a broader panel of rice genotypes tested. This study shows that the amount of zinc in grain did not influence blast severity, but foliar zinc content did. Thus, zinc biofortification in rice grain may not reduce the susceptibility of rice to blast. Furthermore, foliar zinc application did not induce resistance but reduced blast disease severity on four susceptible rice genotypes. Screening for biotic, abiotic stresses and yield potential before genotype release is also key. Most of the zinc-biofortified varieties were resistant to blast but the yield was lower than that of the local checks (Komboka, ARICA 17 and BW196). These findings show that the zinc-biofortified genotypes were not agronomically superior to the contemporary rice varieties, and there is a need to apply genomic selection to combine promising alleles into adapted rice genetic backgrounds. With rice becoming a staple food for most Kenyans, the goal of the IRRI-KALRO rice biofortification effort is to boost the nutritional value of rice through breeding for substantial zinc into high-yielding rice varieties.

## Figures and Tables

**Table 1:** Response of rice genotypes after inoculation with two isolates of *Magnaporthe oryzae* from Kenya.

Rice Genotype	* Binary Reaction to Isolate		Foliar Disease Severity (0–9 Scale)		
	KE0002	KE0215	Mean Severity ± SE	Confidence Limits (95%)	
				Lower	Upper
BASMATI 370	S	S	5.6 ± 0.2 A	5.18	5.93
IRBLK-KA	R	S	4.7 ± 0.2 AB	4.29	5.04
IR 96248-16-2-3-3-B	S	S	4.5± 0.2 B	4.09	4.85
IRBL3-CP4	S	R	2.3 ± 0.2 C	2.15	2.91
IRBL11-ZH	R	S	2.3 ± 0.2 C	1.96	2.71
IRBLTA 2-RE	R	R	1.3 ± 0.2 D	0.96	1.71
IRBTP16211 (LTH)	R	R	1.3 ± 0.2 D	0.87	1.63
IR 99681-38-1-2	R	R	1.1± 0.2 D	0.76	1.52
IR 99639-81-3-2	R	R	1.1 ± 0.2 D	0.76	1.52
BW196	R	R	1.1 ± 0.2 D	0.71	1.46
IR10M210	R	S	1.1 ± 0.2 D	0.71	1.46
IR99674-25-3-2	R	R	1.1 ± 0.2 D	0.68	1.43
IRAT 109	R	R	1.0 ± 0.2 D	0.65	1.41
IR 99674-60-2-1	R	R	1.0 ± 0.2 D	0.62	1.38
IR 99636-93-3-3	R	S	1.0 ± 0.2 D	0.59	1.35
Komboka	R	R	1.0 ± 0.2 D	0.57	1.32
IR08M118	R	R	0.9 ± 0.2 D	0.57	1.32
ARICA 17	R	R	0.9 ± 0.2 D	0.54	1.29
IR 99647-26-1-3	R	R	0.9 ± 0.2 D	0.54	1.29
IR 99637-123-1-3	R	R	0.8 ± 0.2 D	0.40	1.16
IR 99636-96-1-3	R	R	0.8 ± 0.2 D	0.40	1.16
IR 97454-60-3-1-B	R	R	0.8 ± 0.2 D	0.37	1.13
IR 99639-33-4-2	R	R	0.7 ± 0.2 D	0.34	1.10

* Binary coding of disease score severity where 0–3 stands for resistance (R), while 4–9 stands for susceptible (S) reactions. Blast severity means followed by the same letter do not differ significantly (Tukey’s HSD, *α* = 0.05). SE is the standard error.

**Table 2:** A comparison of the zinc content in the grain and leaves among the rice panel which had been inoculated with two isolates of *Magnaporthe oryzae*.

GENOTYPE	Zinc in the Grain			Zinc in the Leaves		
	^1^ PPM	^2^ BLUP	RANK	^3^ PPM	BLUP	RANK
ARICA 17	20.00	−0.1097 ns	5	196.83	59.0923 ***	18
BASMATI 370	21.00	0.1595 ns	12	63.16	−34.8033 ***	2
BW196	21.00	0.1595 ns	12	137.55	1.3993 ns	11
IR08M118	20.33	−0.0199 ns	7	119.5	−31.5948 **	4
IR10M210	21.00	0.1595 ns	12	85.52	−6.5909 ns	10
IR 96248-16-2-3-3-B	21.00	0.1595 ns	12	109.36	−21.1050 *	6
IR 97454-60-3-1-B	19.67	−0.1994 ns	1	264.02	37.2548 ***	15
IR 99636-93-3-3	20.67	0.0698 ns	11	67.03	−67.2362 ***	1
IR 99636-96-1-3	19.67	−0.1994 ns	1	173.17	−15.5868 ns	8
IR 99637-123-1-3	21.67	0.3389 ns	17	209.27	38.0934 ***	16
IR 99639-33-4-2	20.00	−0.1097 ns	5	125.48	57.06815 ***	17
IR 99639-81-3-2	20.33	−0.0199 ns	7	133.82	−15.9745 ns	7
IR 99647-26-1-3	19.67	−0.1994 ns	1	87.8	6.03515 ns	12
IR99674-25-3-2	20.33	−0.1994 ns	1	141.25	−29.7181 **	5
IR 99674-60-2-1	20.33	−0.0199 ns	7	84.77	−33.0060 **	3
IR 99681-38-1-2	19.67	−0.0199 ns	7	105.58	−8.9348 ns	9
IRAT 109	21.00	0.1595 ns	12	169.1	32.1048 **	14
IRRI 215	20.00	−0.1097 ns	5	170.05	15.3992 ns	13

^1^ Zinc concentrations in grain, parts per million (ppm) analyzed from rice which was grown under field conditions in the main season at Mwea, Kenya; ^2^ BLUPs are the best linear unbiased predictors of zinc content. *, **, *** means the BLUPs differed significantly (Tukey’s HSD, *alpha* = 0.05, 0.01 and <0.001, respectively); BLUPs followed by ns were not significant in the model; ^3^ zinc content was analyzed on leaves of plants which had been grown in the screenhouse.

**Table 3:** Blast severity in susceptible rice genotypes which had foliar zinc of varying concentrations applied and were inoculated with two isolates of *Magnaporthe oryzae*.

Rice Genotype	Disease Severity (0–9 Scale)			Binary Disease Reaction
	Mean ± SE	Confidence Limit (95%)		
		Lower	Upper	
IR96248-16-2-3-3-B	8.28 ± 0.23 A	7.82	8.74	S
IRBLK-KA	7.56 ± 0.23 A	7.10	8.01	S
BASMATI 370	6.67 ± 0.23 B	6.21	7.13	S
BASMATI 217	5.7 ± 0.28 C	5.26	6.18	S

Blast severity was scored on a 0–9 scale, where 0 to 3 represented disease resistance (R) as expressed by varying levels of hypersensitive reaction and 4 to 9 represented susceptibility (S) with increasing lesion size. Severity means followed by the same letter do not differ significantly (Tukey’s HSD, *alpha* = 0.05).

**Table 4:** Blast severity in susceptible rice cultivars which were sprayed with varying concentrations of zinc solution and inoculated with two blast pathogen isolates.

Zinc in Foliar Fertilizer (%)	Disease Severity (0–9 Scale)			Binary Disease Reaction
	Mean ± SE	Confidence Limits (95%)		
		Lower	Upper	
0	7.67 ± 0.28 A	7.10	8.23	S
0.07	7.25 ± 0.28 AB	6.69	7.81	S
0.05	7.17 ± 0.28 AB	6.60	7.73	S
0.105	7.17 ± 0.28 AB	6.60	7.73	S
0.15	6.58 ± 0.28 AB	6.02	7.15	S
0.2	6.50 ± 0.28 B	5.94	7.06	S

Blast severity was scored on a 0–9 scale, where 0 to 3 represented disease resistance (R) as expressed by varying levels of hypersensitive reaction and 4 to 9 represented susceptibility (S) with increasing lesion size. Severity means followed by the same letter do not differ significantly (Tukey’s HSD, *alpha* = 0.05.

**Table 5:** Comparison of agronomic traits (days to flowering, yield (t ha^−1^), plant height (cm), panicle length (cm), panicle number, tiller number, thousand-grain weight (g) and panicle blast severity) for rice genotypes evaluated in the field without zinc foliar application.

Genotype	Days to Flowering ± SE	Yield (t ha^−1^) ± SE	Plant Height (cm) ± SE	Panicle Length (cm) ± SE	Panicle Number ± SE	Tiller Number ± SE	1000-Grain Weight (g) ± SE	Panicle Blast Severity ± SE
BW196	97.00 ± 2.23 A	5.95 ± 0.32A	98.20 ± 4.31 B	20.27 ± 0.81 A	25.93 ± 2.34 A	30.20 ± 2.02 A	28.11 ± 1.19 A	0.00 ± 0.44 D
IRAT 109	92.00 ± 2.23 AB	2.57 ± 0.32 DEFG	96.53 ± 4.31 B	20.67 ± 0.81 A	15.93 ± 2.34 AB	18.80 ± 2.02 B	23.56 ± 1.19 AB	0.67 ± 0.44 CD
IR 99636-96-1-3	88.00 ± 2.23 ABC	2.70 ± 0.32 CDEFG	98.60 ± 4.31 B	19.27 ± 0.81 A	13.60 ± 2.34 AB	14.47 ± 2.02 B	23.22 ± 1.19 AB	0.00 ± 0.44 D
IR99674-25-3-2	87.67 ± 2.23 ABC	2.47 ± 0.32 DEFG	96.53 ± 4.31 B	20.33 ± 0.81 A	12.60 ± 2.34 B	14.87 ± 2.02 B	24.56 ± 1.19 AB	0.33 ± 0.44 D
IR 99681-38-1-2	85.67 ± 2.23 ABCD	3.64 ± 0.32 BCDE	104.60 ± 4.31 AB	21.53 ± 0.81 A	19.13 ± 2.34 AB	18.07 ± 2.02 B	21.45 ± 1.19 B	0.00 ± 0.44 D
IR10M210	84.33 ± 2.23 BCD	3.69 ± 0.32 BCDE	103.13 ± 4.31 AB	20.13 ± 0.81 A	16.07 ± 2.34 AB	17.80 ± 2.02 B	23.56 ± 1.19 AB	3.00 ± 0.44 BC
Komboka	84.33 ± 2.23 BCD	4.99 ± 0.32 AB	101.33 ± 4.31 AB	20.73 ± 0.81 A	15.00 ± 2.34 AB	18.93 ± 2.02 B	23.22 ± 1.19 AB	1.00 ± 0.44 CD
IR 99637-123-1-3	84.00 ± 2.23 BCD	3.05 ± 0.32 CDEF	92.93 ± 4.31 B	22.20 ± 0.81 A	18.00 ± 2.34 AB	18.73 ± 2.02 B	21.44 ± 1.19 B	1.00 ± 0.44 CD
IR 99639-33-4-2	83.67 ± 2.23 BCD	2.12 ± 0.32 DEFG	110.07 ± 4.31 AB	22.40 ± 0.81 A	17.87 ± 2.34 AB	18.93 ± 2.02 B	19.56 ± 1.19 B	0.00 ± 0.44 D
IR 99674-60-2-1	83.00 ± 2.23 BCD	2.73 ± 0.32 CDEFG	101.13 ± 4.31 AB	20.33 ± 0.81 A	15.60 ± 2.34 AB	18.40 ± 2.02 B	23.44 ± 1.19 AB	0.00 ± 0.44 D
IR 99647-26-1-3	82.67 ± 2.23 BCD	2.66 ± 0.32 DEFG	100.13 ± 4.31 AB	21.07 ± 0.81 A	16.27 ± 2.34 AB	18.27 ± 2.02 B	22.67 ± 1.19 AB	0.00 ± 0.44 D
IR08M118	82.00 ± 2.23 BCD	3.81 ± 0.32 BCD	107.53 ± 4.31 AB	21.87 ± 0.81 A	15.20 ± 2.34 AB	18.27 ± 2.02 B	23.67 ± 1.19 AB	0.00 ± 0.44 D
IR 99636-93-3-3	82.00 ± 2.23 BCD	2.04 ± 0.32 EFG	105.00 ± 4.31 AB	21.20 ± 0.81 A	19.47 ± 2.34 AB	21.60 ± 2.02 AB	20.67 ± 1.19 B	2.33 ± 0.44 CD
IR 96248-16-2-3-3-B	81.67 ± 2.23 BCD	1.79 ± 0.32 FG	122.27 ± 4.31 A	20.87 ± 0.81 A	14.40 ± 2.34 AB	16.33 ± 2.02 B	19.22 ± 1.19 B	7.00 ± 0.44 A
IR 99639-81-3-2	81.33 ± 2.23 BCD	2.07 ± 0.32 EFG	98.80 ± 4.31 B	20.87 ± 0.81 A	19.80 ± 2.34 AB	17.60 ± 2.02 B	23.45 ± 1.19 AB	1.00 ± 0.44 CD
IR 97454-60-3-1-B	80.67 ± 2.23 BCD	3.12 ± 0.32 CDEF	95.20 ± 4.31 B	19.67 ± 0.81 A	14.07 ± 2.34 AB	15.67 ± 2.02 B	22.22 ± 1.19 AB	1.00 ± 0.44 CD
IRBL11-ZH	80.33 ± 2.23 BCD	1.53 ± 0.32 FG	107.73 ± 4.31 AB	22.33 ± 0.81 A	19.13 ± 2.34 A B	21.80 ± 2.02 AB	20.44 ± 1.19 B	1.67 ± 0.44 CD
ARICA 17	78.67 ± 2.23 CD	4.40 ± 0.32 ABC	100.13 ± 4.31 AB	21.13 ± 0.81 A	14.73 ± 2.34 AB	18.00 ± 2.02 B	20.89 ± 1.19 B	1.00 ± 0.44 CD
BASMATI 370	78.67 ± 2.23 CD	1.02± 0.32 G	108.60 ± 4.31 AB	20.00 ± 0.81 A	13.67 ± 2.34 AB	17.73 ± 2.02 B	20.22 ± 1.19 B	7.00 ± 0.44 A
IRBTP16211	78.00 ± 2.23 CD	1.99 ± 0.32 EFG	103.40 ± 4.31 AB	22.20 ± 0.81 A	17.47 ± 2.34 AB	20.67 ± 2.02 AB	19.44 ± 1.19 B	1.00 ± 0.44 CD
IRBL3-CP4	76.67 ± 2.23 CD	1.57 ± 0.32 FG	106.00 ± 4.31 AB	19.87 ± 0.81 A	19.20 ± 2.34 AB	21.13 ± 2.02 AB	19.44 ± 1.19 B	5.00 ± 0.44 AB
IRBLK-KA	76.67 ± 2.23 CD	2.16 ± 0.32 DEFG	101.27 ± 4.31 AB	22.93 ± 0.81 A	19.73 ± 2.34 AB	22.47 ± 2.02 AB	21.33 ± 1.19 B	6.33 ± 0.44 A
IRBLTA 2-RE	75.00 ± 2.23 CD	2.50 ± 0.32 DEFG	104.67 ± 4.31 AB	22.60 ± 0.81 A	17.20 ± 2.34 AB	19.00 ± 2.02 B	22.00 ± 1.19 AB	1.67 ± 0.44 CD

Means followed by the same letter do not differ significantly (Tukey’s HSD, *alpha* = 0.05).

**Table 6:** Correlations between blast severity and the agronomic traits of the twenty-three rice genotypes, which were evaluated in the field during the main rice-growing season at Mwea Research Station, Kenya.

Row	Days to Flowering	Yield t/ha	Foliar Blast Severity	Plant Height (cm)	Thousand-Grain Weight (g)	Panicle Number	Tiller Number	Panicle Blast Severity
Days to flowering	1	0.5397; 0.01138	−0.4608; 0.0269	−0.3426; 0.0402	0.7267; 0.0013	0.2125; 0.5064	0.2239; 0.2984	−0.4774; 0.0015
Grain yield t/ha		1	−0.4920; 0.00598	−0.3747; 0.01482	0.6772; 0.0007	0.2271; 0.54387	0.3176; 0.60076	−0.4922; 0.0076
Foliar blast severity			1	0.5402; 0.0361	−0.4115; 0.1484	−0.0643; 0.3023	0.0657; 0.2405	0.9146; 0.0005
Plant height (cm)				1	−0.5567; 0.0023	−0.0676; 0.6830	−0.0208; 0.2863	0.5433; 0.0750
1000-grain weight (g)					1	0.2080; 0.5833	0.2493; 0.3101	−0.4986; 0.0078
Panicle number						1	0.8802; 0.000	−0.0657; 0.7560
Tiller number							1	0.0308; 0.5674

The top figure in a cell represents Spearman’s correlation, and the bottom figure is the *p*-value. Foliar blast severity was scored on 28-day-old seedlings which had been grown in a screenhouse without zinc supplementation. The agronomic traits data were collected at various growth stages of the twenty-three rice genotypes. Panicle blast severity was scored on plants which had been grown and naturally infected by the resident field blast pathogen at Mwea. Blast severity was scored on a scale of 0–9, where 0 = no visible damage on the leaves or panicles; 9 = visible damage on more than 75% of the leaf or panicle tissue. Zinc content in the leaves was analyzed on a sub-set of the twenty-three rice genotypes of 21-day-old seedlings which had not been inoculated with blast isolates.

**Table 7:** Description of the rice panel (n = 23) that was inoculated with *Magnaporthe oryzae*.

Rice Genotype	Parentage	^1^ *Pi* Gene	Description
IR 99636-96-1-3	IR 83317-AC 124/IR 69428-6-1-1-3-3//IR 83317-AC 71/IR 68144-2B-2-2-3-1-166///IR 36	Unknown	Zinc-biofortified
IR99674-25-3-2	PAU 3105-45-3-2/IR 82802-36-3-3-1-3///IR 91153-AC 82/IR05F102//IR 68144-2B-2-2-3-1-166/6/PR 115/IR01W102/5/BR 802-118-4-2 (BRRI DHAN 29)/IR 68144-2B-2-2-3-1-166//IR 69428-6-1-1-3-3///IR 64/4/IR 70114-5-3-3-3	Unknown	Zinc-biofortified
IR 99681-38-1-2	BR 29*2/IR 69428-6-1-1-3-3	Unknown	Zinc-biofortified
IR10M210	IRRI 123/IR 68144-2B-2-2-3-1-127	Unknown	Zinc-biofortified
IR 99637-123-1-3	IR 68144-2B-2-2-3-1-166/IR05N496//IR 75862-206-2-8-3-B-B-B/IR05N496///IR 83317-AC 15/IR 68144-2B-2-2-3-1-166//IR 83317-AC 71/IR 68144-2B-2-2-3-1-166	Unknown	Zinc-biofortified
IR 99639-33-4-2	IR 83317-AC 116/IR 83317-AC 80//IR 91143-AC 243/IR 83317-AC 25///IR 64	Unknown	Zinc-biofortified
IR 99674-60-2-1	PAU 3105-45-3-2/IR 82802-36-3-3-1-3///IR 91153-AC 82/IR05F102//IR 68144-2B-2-2-3-1-166/6/PR 115/IR01W102/5/BR 802-118-4-2 (BRRI DHAN 29)/IR 68144-2B-2-2-3-1-166//IR 69428-6-1-1-3-3///IR 64/4/IR 70114-5-3-3-3	Unknown	Zinc-biofortified
IR 99647-26-1-3	IR 91152-AC 443/IR 66//IR 91152-AC 438/BR 29	Unknown	Zinc-biofortified
IR08M118	IR 69092-57-3/IRRI 123	Unknown	Zinc-biofortified
IR 99636-93-3-3	IR 83317-AC 124/IR 69428-6-1-1-3-3//IR 83317-AC 71/IR 68144-2B-2-2-3-1-166///IR 36	Unknown	Zinc-biofortified
IR 96248-16-2-3-3-B	IR07F289/2*IR 69428-6-1-1-3-3//IR09N481///IR03A568	Unknown	Zinc-biofortified
IR 99639-81-3-2	IR 83317-AC 116/IR 83317-AC 80//IR 91143-AC 243/IR 83317-AC 25///IR 64	Unknown	Zinc-biofortified
IR 97454-60-3-1-B	NSIC RC 158/NEGRO//BR 29	Unknown	Zinc-biofortified
IRBL11-Zh	LIJIANG XINTUAN HEIGU (AC 59323)*3/ZHAI YE QING 8	*Pi-11*	Blast disease differential line
IRBTP16211 (LTH)	LIJIANG XINTUAN HEIGU (AC 59323)	*Pik-1*	Recurrent parent for blast differential lines
IRBL 3-CP 4	LIJIANG XINTUAN HEIGU (AC 59323)*3/C 104 PKT	*Pi-3*	Blast disease differential line
IRBL K-KA	LIJIANG XINTUAN HEIGU (AC 59323)*3/Kanto 51	*Pik*	Blast disease differential line
IRBLTA 2-RE	LIJIANG XINTUAN HEIGU (AC 59323)*3/Reiho	*Pita-2*	Blast disease differential line
ARICA 17	Scrid017-1-4-4-4-1	Unknown	A variety cultivated in the highlands of Ethiopia
BW196 (NIBAM 109)	K 8 (NATURAL MUTANT)/.//HONDARAWALA/C 104	Unknown	A variety cultivated in Kenya and Tanzania
IRAT 109	IRAT 13/IRAT 10	Unknown	A variety cultivated in Kenya and Tanzania
BASMATI 370	NIBAM 11	Unknown	A popular blast susceptible aromatic variety cultivated in East Africa
R05N221 (also called Komboka)	IR 74052-297-2-1/IR 71700-247-1-1-2	Unknown	A variety cultivated in Kenya and Tanzania

^1^ Based on the information which was reported about the Pi genes that were introgressed in the international rice blast differential [36]. Unknown means that the current work was conducted without prior knowledge of the effective Pi genes in the backgrounds of the rice genotypes listed herein.

## Data Availability

The raw data validating the conclusions in this manuscript will be made available on request from the authors. The data are not publicly available due to the nature of collaborative agreement on data sharing.
